# Increased efficiency in identifying mixed pollen samples by meta-barcoding with a dual-indexing approach

**DOI:** 10.1186/s12898-015-0051-y

**Published:** 2015-07-22

**Authors:** Wiebke Sickel, Markus J Ankenbrand, Gudrun Grimmer, Andrea Holzschuh, Stephan Härtel, Jonathan Lanzen, Ingolf Steffan-Dewenter, Alexander Keller

**Affiliations:** Department of Animal Ecology and Tropical Biology, Biocenter, University of Würzburg, Am Hubland, 97074 Würzburg, Germany

**Keywords:** DNA barcoding, High throughput sequencing, Illumina MiSeq platform, ITS2, Next generation sequencing, NGS, *Osmia*, Palynology, Pollination ecology

## Abstract

**Background:**

Meta-barcoding of mixed pollen samples constitutes a suitable alternative to conventional pollen identification via light microscopy. Current approaches however have limitations in practicability due to low sample throughput and/or inefficient processing methods, e.g. separate steps for amplification and sample indexing.

**Results:**

We thus developed a new primer-adapter design for high throughput sequencing with the Illumina technology that remedies these issues. It uses a dual-indexing strategy, where sample-specific combinations of forward and reverse identifiers attached to the barcode marker allow high sample throughput with a single sequencing run. It does not require further adapter ligation steps after amplification. We applied this protocol to 384 pollen samples collected by solitary bees and sequenced all samples together on a single Illumina MiSeq v2 flow cell. According to rarefaction curves, 2,000–3,000 high quality reads per sample were sufficient to assess the complete diversity of 95% of the samples. We were able to detect 650 different plant taxa in total, of which 95% were classified at the species level. Together with the laboratory protocol, we also present an update of the reference database used by the classifier software, which increases the total number of covered global plant species included in the database from 37,403 to 72,325 (93% increase).

**Conclusions:**

This study thus offers improvements for the laboratory and bioinformatical workflow to existing approaches regarding data quantity and quality as well as processing effort and cost-effectiveness. Although only tested for pollen samples, it is furthermore applicable to other research questions requiring plant identification in mixed and challenging samples.

**Electronic supplementary material:**

The online version of this article (doi:10.1186/s12898-015-0051-y) contains supplementary material, which is available to authorized users.

## Background

Identification of pollen origin is a central aspect in pollination ecology studies [1–3] and agro-ecological research [4, 5]. Conventional pollen identification utilises light microscopy and discriminates species according to morphological characteristics [6]. This requires expert knowledge for the bioregion and taxa of interest [7], is time-consuming [8] and lacks discriminatory power at lower taxonomic levels [4, 8].

**Figure 1:**
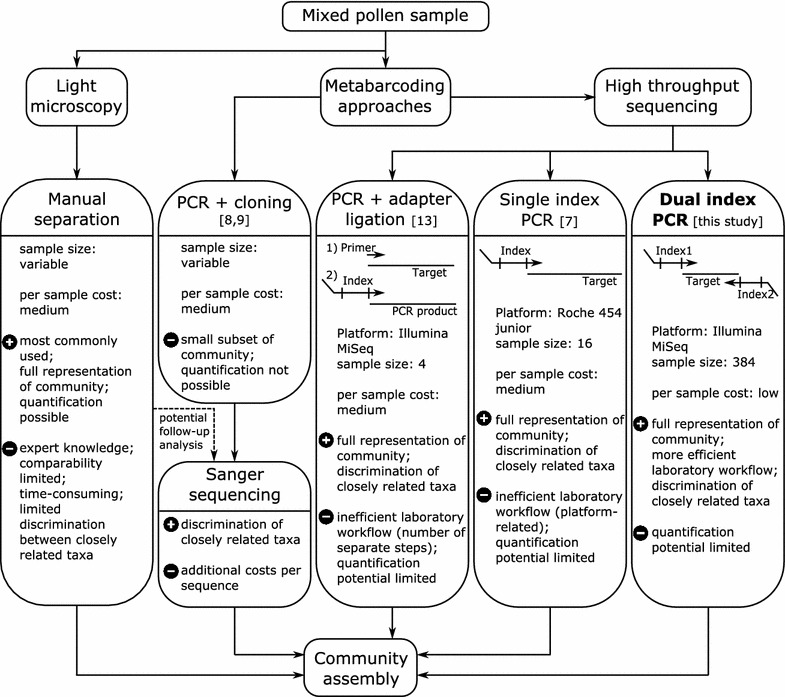
Comparison of different approaches for plant species identification in mixed pollen samples.

A promising approach to circumvent these issues has been to identify plant species in pollen samples by DNA sequence analysis. This can be done by, for example, cloning amplified PCR products into plasmids and sequencing a subset of clones [8, 9] or sequencing pollen grains of interest [10, 11] or bee crop contents directly [12]. However, this often does not reflect the complete diversity of plant species present, since only a subset of DNA sequences are analysed or only dominant plant taxa can be detected. Recent studies [7, 13–15] have identified high throughput sequencing (HTS) approaches based on meta-barcoding as a suitable alternative for existing methods. However, current protocols still suffer from a limited sample throughput [7, 14, 15] and/or practicability issues due to separate steps for PCR amplification and index labelling [13]. We here present a protocol for highly multiplexed pollen sequencing utilising a dual-indexing strategy [16]. An overview of existing methods alongside our new approach is given in Figure 1. We designed meta-barcoding primers suitable for plant identification using the internal transcribed spacer 2 (ITS2) that already incorporate Illumina-specific adapters for high-throughput sequencing as well as new sequencing primers that are added to the sequencing flow cell. The rationale for using ITS2 rather than other genetic markers for plant DNA barcoding in general is provided elsewhere [17] and its applicability regarding meta-barcoding criteria has also been successfully demonstrated [7, 13]. We tested our new approach by sequencing 384 pollen samples collected by two solitary bees species with known different foraging strategies: polylectic *Osmia bicornis* [18] and oligolectic *Osmia truncorum* [19]. Alongside this enhancement of the laboratory method, we updated the reference database used for ITS2 meta-barcoding [7] and added compatibility for the UTAX classification software [20] as a second and alternative strategy beside the RDP classifier [7, 21].

## Methods

### Dual-indexing design

As amplifying primers we used the well-established combination of plant barcoding primers ITS-S2F [17] and ITS4R [22]. These were already used for plant species identification based on meta-barcoding [7] and deliver a fragment of suitable size for MiSeq v2 sequencing using 500 cycles. For MiSeq-conformity, we expanded each of the primers according to the overall oligo scaffold described in Kozich et al. [16]. This scaffold consists of MiSeq-specific adapters, an 8nt index sequence, a 10nt pad as well as a 2nt linker sequence and lastly the amplifying primers. To successfully transfer the scaffold design to ITS2 sequencing, we ensured by minor modifications that the melting temperature (T_m_) of the combined pad, linker and amplifying primer was ~65°C (see Additional file of Kozich et al. [16]) enabling the read primers to bind during the later sequencing procedure. In the forward scaffold, we adapted the pad sequence from 5′-$$\tt{TATGGTAATT}$$-3′ to 5′-**CC**$$\tt{TGGT}$$**GC**$$\tt{T}$$**G**-3′ (adapted nucleotides in bold). The pad of the reverse scaffold remained unchanged. Complete sequences of the final oligos were forward: 5′-$$\tt{AATGATACGGCGACCACCGAGATCTACAC XXXXXXXX}$$**CC**$$\tt{TGGT}$$**GC**$$\tt{T}$$**G**$$\tt{GT}$$**ATGCGATACTTGGTGTGAAT**-3′ and reverse: 5′-$$\tt{CAAGCAGAAGACGGCATACGAGAT}$$$$\tt{XXXXXXXX}$$$$\tt{AGTCAGTCAG}$$$$\tt{CC}$$**TCCTCCGCTTATTGATATGC**-3′, where adapted nucleotides are denoted in bold and $$\tt{XXXXXXXX}$$ indicates the index sequences used for multiplexing. Both primer sequences were thus 32nt long, had a T_m_ of 64.8°C, a 50% GC content and exhibited low self-complementarity (longest dimer complement: 4 bp). They amplify a total fragment of approximately 470–480 bp, including the complete ITS2 sequence. The actual sequenced part of this fragment covers 350–360 bp (target only) and is thus within the range of 2 × 250 cycles sequencing, leaving some buffer for joining the paired end reads. We used 16 forward index sequences SA501–SB508 and 24 reverse indices SA701–SB712, allowing a total of 384 unique combinations for sample indexing (Additional file of Kozich et al. [16]). With ITS2-specific modifications, it was also necessary to modify the sequencing primers that are added to the MiSeq flow cell. We thus changed read and index primers as follows (adapted nucleotides in bold): Read1: 5′-**CC**$$\tt{TGGT}$$**GC**$$\tt{T}$$**G**$$\tt{GT}$$**ATGCGATACTTGGTGTGAAT**-3′, Read2: 5′-$$\tt{AGTCAGTCAG}$$$$\tt{CC}$$**TCCTCCGCTTATTGATATGC**-3′, Index: 5′-**GCATATCAATAAGCGGAGGA**$$\tt{GG}$$$$\tt{CTGACTGACT}$$-3′.

### Processing test samples

The newly designed dual-indexing approach was evaluated with mixed pollen samples, collected from nests of the solitary bees *Osmia bicornis* (270 samples), *Osmia truncorum* (111 samples) and other *Osmia spp*. (3 samples) at various sites near Würzburg, Germany from April to September 2013. Different samples originated from pools of two different brood cells from the same nest (likely the same mother bee few days apart). We chose this study system because we wanted to demonstrate that different foraging strategies can be detected using pollen meta-barcoding. We documented flower resources available during the sample period within a 50 m radius (all plant species) and within a 600 m radius (mass-flowering plants only) around the nest sites. This was done to gain information on species identity of flower resources available for bee foraging at the time of sampling (Additional file 1) and to be able to compare them with our sequence data.

DNA from ~0.003 g pollen grains was isolated as described by Keller et al. [7] using the Macherey-Nagel Food Kit (Düren, Germany). PCR was performed in three separate 10 µL reactions in order to avoid PCR bias [23]. Each reaction contained 5 µL 2 × Phusion Master Mix (New England Biolabs, Ipswich, MA, USA), 0.33 µM each of the forward and reverse primers, 3.34 µL PCR grade water and 1 µL DNA template. PCR conditions were as follows: initial denaturation at 95°C for 4 min, 37 cycles of denaturation at 95°C for 40 s, annealing at 49°C for 40 s and elongation at 72°C for 40 s; followed by a final extension step at 72°C for 5 min. Each sample was assigned a different forward/reverse index combination for sample-specific labelling. Triplicate reactions of each sample were combined after PCR and further processed as described in Kozich et al. [16], including between-sample normalization using the SequalPrep™ Normalization Plate Kit (Invitrogen GmbH, Darmstadt, Germany) and pooling of 96 samples. These pools were quality controlled using a Bioanalyzer High Sensitivity DNA Chip (Agilent Technologies, Santa Clara, CA, USA), quantified with the dsDNA High Sensitivity Assay (Life Technologies GmbH, Darmstadt, Germany), and afterwards combined to a single pool containing all 384 samples. This was diluted to 8 pM, denatured and spiked with 5% Phix Control Kit v3 (Illumina Inc., San Diego, CA, USA) according to the Sample Preparation Guide (llumina Inc. 2013). Sequencing was performed on the Illumina MiSeq using 2 × 250 cycles v2 chemistry (Illumina Inc., San Diego, CA, USA).

### Data analysis

Raw sequence reads were obtained from the Illumina MiSeq output directly, which includes sample reads already demultiplexed by the MiSeq Reporter v. 2.5.1.3 with perfect index matches only. Forward and reverse reads were joined using the join_paired_ends.py command in QIIME v.1.8.0 [24] using default parameters. Low quality reads were removed (<Q20, <150 bp, ambiguous base-pairs) with USEARCH v8.0.1477 [25]. Combined reads were taxonomically classified with the RDP classifier [21] as well as the UTAX algorithm and results compared to show that the data is compatible between both alternative analytical strategies. UTAX and RDP were executed for each sample separately.

In the following, we concentrate on UTAX, since the RDP classifier has been used previously for pollen taxonomic assignments [7]. A raw score cut-off at 20 was used, as the UTAX algorithm does currently not provide bootstrap comparable confidence values (but is expected to incorporate these soon, see http://drive5.com/usearch/manual/faq_taxconfs.html, accessed 2015/22/05). These assignment scores are however comparable between reads as long as subsequent analyses do base all upon the same database.

For data analysis, the raw UTAX output was parsed using a self-written perl script, which counts the number of assignments for each taxon and aggregates these into a single table (https://github.com/iimog/meta-barcoding-dual-indexing). This table is converted into a community matrix format, with rows as species and columns representing samples, and a separate file with the taxonomic lineage of each species is also created. These files are directly importable into common statistical software, e.g. *R* v.3.1.2 [26] using the package *phyloseq* v.1.6.1 [27]. To assess sufficiency of the sequencing depth, we created species accumulation curves for each sample using the *vegan* package v2.2-0 [28] in *R* v.3.1.2 [26], excluding taxa accounting for less than 0.1% of sample reads. Additionally, we determined the ten most abundant plant families collected by *O. bicornis* and *O. truncorum*.

### Reference database update

Beside the enhancement of the laboratory protocol, we considered it important to address also the actuality and completeness of the reference database. We thus performed an update according to the annotation pipeline described for the ITS2 database [29, 30]. For this, we extracted all available ITS2 sequences belonging to Viridiplantae from GenBank [31] (accessed on 2015/19/01) as described in detail in Koetschan et al. [30]. The taxonomy follows the NCBI taxonomy database [32], which may not perfectly reflect evolutionary status, but is well usable for automatic procedures, due to its integration into the public NCBI framework. Taxonomy was assigned to the sequences by mapping the gi to the NCBI taxid. Taxonomic levels were selected at seven levels (kingdom, phylum, class, order, family, genus, species) using a custom perl script utilizing the NCBI::Taxonomy module by courtesy of F. Förster (doi:10.5281/zenodo.17375). RDP training files, a UTAX database and taxtree were created with a custom perl script (https://github.com/iimog/meta-barcoding-dual-indexing). The database update, scripts and information on how to use it with the RDP classifier or UTAX are provided at http://www.dna-analytics.biozentrum.uni-wuerzburg.de.

## Results

### Sequencing output and data analysis

**Figure 2:**
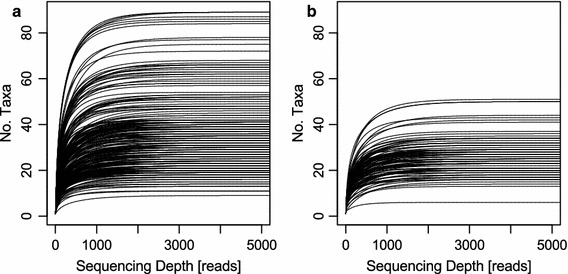
Species accumulation curves. **a** *Osmia bicornis* samples; **b** *Osmia truncorum* samples. The x-axis was limited to 5,000 reads as the saturation of all samples was below this threshold. The y-axis was limited to 90 taxa in both plots to obtain the same scale. Taxa accounting for less than 0.1% of total sample reads were excluded.

In total we obtained 11,624,087 raw ITS2 reads (PhiX excluded), which accounted for an average of 30,271 [standard deviation (SD): 11,373; median: 30,900] reads per sample. After data processing (low-quality <Q20, short reads <150 bp, ambiguous base-pairs), a mean of 15,580 (SD 6,598; median 15,740) reads per sample remained. Species accumulation curves (Figure 2) show that almost all samples were sequenced to saturation after approximately 2,000–3,000 high quality reads. Based on the ratio of raw to high quality reads, this accounts for approximately 4,000–6,000 raw reads required. Per sample pollen in bee brood cells originated from between one and 85 different plant species (Figure 2). Five per cent of samples (19) yielded an output of less than 2,000 reads (minimum saturation threshold, Figure 2), which were removed prior to further analysis. Raw sequences are accessible via the EBI-SRA with the project accession number PRJEB8640.

### Reference database update

Our previously published database contained 73,853 reference sequences of 37,403 unique plant species [7]. The updated version now contains 182,505 plant sequences from 72,325 different species. This is an increase by factor 2.47 (147% additional) for sequences and 1.93 (93% additional) for unique species. In comparison with the original reference set [7], with these data 80.1% (original 53.1%) of the plant species and 90.4% (original 75%) of the genera in Bavaria, Germany, where our test samples originate from, were covered (data retrieved from http://bayernflora.de; accessed on 2015/01/24). Correspondingly, for plant species in the USA, the database covers 66.5–79.1% (median 76.1%) of species and 73.8–87.3% (median 84.9%) of genera, depending on the US state (data retrieved from the BISON project; http://bison.usgs.ornl.gov; accessed on 2015/04/02). In both cases, Bavaria and USA, missing species are likely rare or endemic to specific regions. A comparison of numbers of genera per order covered in the old and updated database versions can be found in the Additional file 2: Table S1.

### Test samples

Regarding our samples, taxonomic classification (after filtering out rare taxa below 0.1%) identified 650 different plant taxa, of which 617 could be classified taxonomically to plant species level, belonging to 288 genera, 71 families, 37 orders and nine classes. The remaining 33 taxa (5%) could not be classified at the species level. Of these, 17 taxa could still be classified at genus level and another seven at the family level. Nine taxa remained that could not be classified even to family level. These belonged to the Sapindales, Fagales and Microthamniales (one taxon each) or remained unclassified (six taxa). At the genus level, RDP and UTAX taxonomic assignments agreed in ~90% of all read classifications, thus both classifiers yielded comparable results.

**Figure 3:**
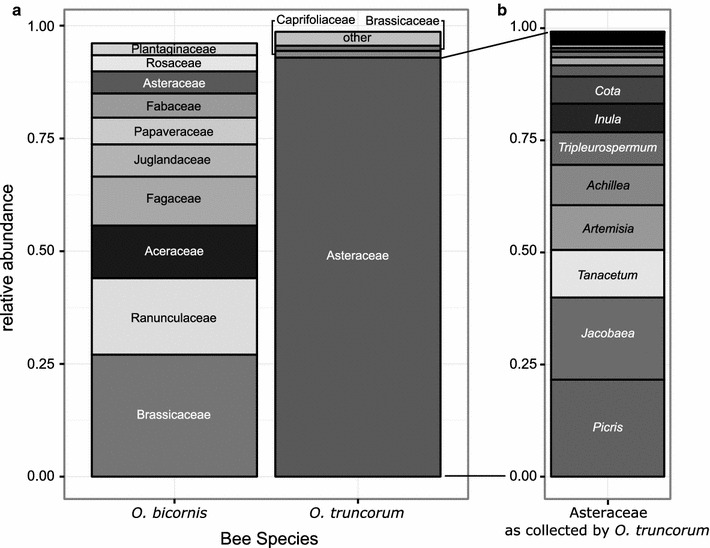
Pollen spectrum of the two bee species. **a** Ten most abundant families as collected by the bee species *O. bicornis* and *O. truncorum*. For *O. truncorum* ‘other’ include the families Apiaceae, Rosaceae, Fabaceae, Ranunculaceae, Plantaginaceae, Juglandaceae and Amaranthaceae. **b** Plant genera detected within the Asteraceae collected by *O. truncorum*. For visualisation reasons, only the eight most abundant genera are labelled. Please note that Aceraceae is now included within Sapindaceae.

For both *Osmia* species together, approximately 50% of documented plant genera (<50 m: all plants, 50–600 m: only mass-flowering plants) were detectable within the sequencing data and contributed with ~75% to all quality-filtered reads. The two bee species differed clearly in foraging patterns as visible through plant families predominantly collected (Figure 3), as well as in the number of plant species with *O. bicornis* collecting up to 85 plant species and *O. truncorum* collecting up to 50 plant species per brood cell (Figure 2). The ten most abundant plant families collected by *O. bicornis* were Brassicaceae (27.07%), Ranunculaceae (16.98%), Aceraceae (11.62%), Fagaceae (10.86%), Juglandaceae (7.16%), Papaveraceae (5.91%) Fabaceae (5.40%), Asteraceae (4.89%), Rosaceae (3.59%) and Plantaginaceae (2.62%). *O. truncorum* pollen was dominated by Asteraceae (92.92%), and only Caprifoliaceae (1.51%) and Brassicaceae (1.14%) contributed more than 1% to the overall collection. The Asteraceae collected by *O. truncorum* contained a wide spectrum of plant genera, with 58 genera being detected, the ten most abundant of which were *Picris, Jacobaea, Tanacetum, Artemisia, Achillea, Tripleurospermum, Inula, Cota, Leucanthemum* and *Crepis* (Figure 3).

## Discussion

High throughput sequencing (HTS) has been shown to be successful and valuable for taxonomic assessment of mixed pollen samples [7, 13, 15]. The drawbacks of existing protocols were the low number of samples processed simultaneously or inefficient multistep library preparations. Recent developments in sequencing technologies allow far larger multiplexing, given the enormous throughput already available with desktop NGS devices. Highly multiplexed sample processing has already been established for bacterial assessments using dual-indexing approaches with the MiSeq sequencer [16]. It was the goal of this study to transfer this knowledge to the field of plant meta-barcoding, in our specific case of pollen samples.

By adapting the primer design to the ITS2 region, modifying the oligo scaffold design, and adjusting the sequencing primers to be compatible with the MiSeq device, we successfully established a fast pollen DNA meta-barcoding routine with high multiplexing capabilities. For our test samples, the newly designed primers were used to sequence 384 mixed pollen samples collected by solitary bees with a single sequencing run. In the original bacterial dual-indexing protocol [16], the potential for higher multiplex rates than 384 samples is suggested depending on required throughput to assess the diversity. Our sequencing results indicate that for pollen samples at least a depth of 2,000–3,000 high quality reads per sample should be reached to identify all taxa within the sample (plateau reached, Figure 2), which was comparable for the two bee species under study. However, this is of course highly dependent on number of plant species in the samples, which may be dependent on sample origin, foraging behaviour and the biodiversity of the ecosystem of interest, but may serve nonetheless as a guideline for higher multiplex rates. Additional index combinations for more samples are provided in the Additional files alongside the protocol for the bacterial dual-index approach [16].

Beside our dual-indexing strategy, another HTS-based approach has been recently proposed. There, PCR amplification and index labelling were conducted in separate steps [13], which is time and labour-intensive and introduces a further step where errors may be introduced. In our protocol, PCR amplification and sample indexing occur simultaneously, which is highly practical and requires no special reagents, such as additional expensive library preparation kits or adapter ligation chemicals. In our protocol, the complete workflow accounts for less than USD 20.00 for materials per sample, when processing 384 samples simultaneously. This is much lower than conventional pollen analysis under the light microscope, which can reach several hundred USD per sample.

Most plant taxa detected could be successfully classified using the already shown RDP classifier [7, 21], but also the recently developed UTAX algorithm [25]. Due to the missing confidence values for taxonomic assignments in UTAX version 8.0 (announced for version 8.1, http://drive5.com/usearch/manual/faq_taxconfs.html, accessed 2015/22/05), we compared the classifications to the RDP output as well as the documented flower resources. UTAX and RDP showed high agreement between taxonomic classifications, thus both may be used arbitrarily.

Approximately half of the genera found flowering near the nest sites were detected in the pollen samples. This is attributable to bee foraging preferences, where not all available resources might be used, especially for the oligolectic *O. truncorum*. Secondly, about three quarters of the reads were assigned to plant genera documented near the nesting sites (<50 m: all plant species, 50–600 m: mass-flowering plants only). As bees are expected to forage also further away, the remaining reads are attributable to pollen collected from undocumented plants or misclassifications.

According to our expectation, pollen composition patterns were very different for the oligolectic and the polylectic bee species (Figure 3). *O. truncorum* samples were dominated by Asteraceae, whereas *O. bicornis* samples showed a wide pollen spectrum. Our data correspond to flower preferences and foraging strategies known for these species [18, 19]. This supports the high quality of information obtained by pollen meta-barcoding, as already intensively evaluated in another study [7]. It is noteworthy that even very rare taxa could be detected, which is of special interest in the oligolectic *O. truncorum* and might be overlooked in light microscopy assessment of pollen samples.

We would like to point out that abundance data obtained from molecular approaches should in general be interpreted with care and only as relative abundance (divided by total number of reads in the sample to account for varying library sizes). Contradicting results exist concerning the suitability of pollen meta-barcoding for quantification purposes, with Keller et al. [7] and Kraaijeveld et al. [14] finding a positive significant correlation between genera by light microscopy and meta-barcoding, whilst Richardson et al. [13] were not able to find such a connection. Due to the different steps in the workflow, e.g. dilutions and PCR, biases can be introduced, leading to skewed data and over- or underrepresentation of certain taxa. PCR bias is considered to be a random process and can be accounted for by performing replicate PCR reactions for each sample [23], which are pooled subsequently. We followed this approach in this study likewise to Keller et al. [7] to avoid PCR bias as far as possible. This may explain some of the discrepancy between studies, although a recent study indicated that PCR replicates might not be necessary in pollen meta-barcoding [14]. The reduced amount of individual processing steps of direct indexing, (as performed here and in both studies identifying positive correlation [7, 14]) further reduces additional risks to introduce unwanted effects in comparison with the study using adapter ligation that shows no correlation [13].

In this study, samples of the same bee species show high consistency in abundance patterns of major taxa, which are easily biologically explainable. A good compromise for most studies investigating foraging patterns might be to not use direct count data, but conservatively categorising plant taxa into ‘abundant’ and ‘rare’ based on a threshold, as proposed by Keller et al. [7]. Where more detail is needed, a subset of samples may also be analysed in parallel by light microscopy for evaluation purposes [7, 13, 14].

One major advantage of pollen meta-barcoding is that no expert knowledge on pollen morphology is required for taxonomic assignment. Additionally, species level assignment is possible even for closely related plant taxa. However, successful taxonomic assignment critically depends on the quality of the reference database. Our target marker was the ITS2 region, but other genetic markers might also be considered for plant species identification using meta-barcoding, e.g. *trn*L [14, 15] or *rbcL* plus *trnH*-*psbA* [8, 9]. The described dual indexing approach [16] can also be applied to other genetic markers, provided some considerations are taken into account as described for ITS2 in this study. On the laboratory side of the workflow, firstly target and thereby primer choice should be appropriate for universal amplification and plant species identification based on DNA sequence data. The amplified fragment should be of the appropriate size for the chosen MiSeq sequencing chemistry, e.g. no longer than ~480–490 bp for 2 × 250 v2 sequencing kits, allowing for some overlap between forward and reverse reads. Given these conditions are met, primer design can be performed following the guidelines from Kozich et al. [16] including the required modifications to the various oligonucleotides. However, as mentioned before, successful plant species identification relies to a large degree also on the underlying reference database and bioinformatical classification algorithm. For most alternative markers comprehensive reference databases are currently lacking and thus taxonomic classifications are mainly performed by a BLAST search [33] against sequences downloaded from GenBank [8, 9, 13–15], locally managed alternative databases [9] and/or newly acquired DNA sequences [8, 9]. BLAST searches are based on local alignments that may only use parts of each sequence (e.g. conserved regions) for classification, lack a hierarchy classification procedure and results can be difficult to interpret [7, 17] especially when results show hits for multiple, different taxa. Setting up locally managed databases is time- and labour-intensive a well as costly and makes it difficult to compare independent studies with one another. In the case of the ITS2 region, we benefitted from the already established ITS2 database [30], which contains annotated and trimmed ITS2 sequences from species worldwide and can be publicly accessed, improving overall comparability across studies.

Although Chen et al. [17] reported high identification accuracies with ITS2 as a genetic marker, some plant taxa could not be identified in recent studies on pollen meta-barcoding [7, 13]. These included the families Salicaceae, Lamiaceae [13] and Vitaceae [7] and the genera *Lonicera* [13], *Heracleum*, *Carduus*, *Phacelia*, *Convolvulus* and *Helianthus* [7], although they had been identified with microscopic pollen analysis. In this study, we could detect all of these taxa. Failure to detect these families and genera with DNA sequence data was most likely due to incompleteness of the reference databases in these studies. Richardson et al. [13] used in total only 2,628 reference sequences, that described about half of the locally occurring plant species. In the case of Keller et al. [7], we were able to directly compare the database then (73,853 sequences) and now (182,505 sequences), which revealed that for each of those plant taxa more reference sequences were included after the database update presented here (Additional file 3: Table S2). This explains the positive detection for those plant taxa in this study in contrast to earlier studies and again highlights the importance of a current and comprehensive reference database for meta-barcoding purposes.

Our test samples comprised only pollen samples collected by bees, but in general ITS2 meta-barcoding can be applied to plant identification in other research fields where mixed samples are encountered, such as diet analysis of herbivores [34, 35] and in palaeo-ecology [36–38]. Furthermore, high-throughput DNA analysis of mixed plant samples can also prove valuable in food safety issues [39], honey quality analysis [8, 9] as well as allergen load assessment [14]. For such applications, alteration of the provided protocol for library preparation and sequencing is not needed, although the DNA extraction process may require alternative kits or adapted protocols specific for the material of interest.

## Conclusions

We have successfully transferred a high-throughput technique for bacterial community sequencing to pollen meta-barcoding, which now enables labour- and cost-effective analysis of up to 384 mixed pollen samples simultaneously, thereby omitting drawbacks of previously established methods. We furthermore enhanced the database used for plant taxa identification based on HTS data. Additionally, our method should be easily adaptable to sample analysis of mixed plant origin in other research fields.

## Availability of supporting data

The data set supporting the results of this article are in the EBI-SRA repository, under the project accession number PRJEB8640. Data on regional flora has been retrieved from http://bayernflora.de for Bavaria (accessed on: 2015/01/24) and from http://bison.usgs.ornl.gov/ for the USA (accessed on 2015/04/02). The database update, scripts and information on how to use it with the RDP classifier or UTAX are provided at http://www.dna-analytics.biozentrum.uni-wuerzburg.de and https://github.com/iimog/meta-barcoding-dual-indexing.

## Additional files

Additional file 1: Plant species documented near solitary bee nest sites. Additional file 2:
**Table S1.** Comparison of the number of genera per order for all orders. Additional file 3:
**Table S2.** Comparison of the number of sequences per group for selected taxonomic groups.

## Abbreviations

HTS: high throughput sequencing
ITS2: internal transcribed spacer 2
T_m_: melting temperature

## References

[1] Carvell C, Westrich P, Meek WR, Pywell RF, Nowakowski M (2006). Assessing the value of annual and perennial forage mixtures for bumblebees by direct observation and pollen analysis. Apidologie.

[2] Köppler K, Vorwohl G, Koeniger N (2007). Comparison of pollen spectra collected by four different subspecies of the honey bee Apis mellifera. Apidologie.

[3] Behl M, Horn H, Schwabe A (2008). Analysis of pollen loads in a wild bee community (Hymenoptera: Apidae)—a method for elucidating habitat use and foraging distances. Apidologie.

[4] Williams NM, Kremen C (2007). Resource distributions among habitats determine solitary bee offspring production in a mosaic landscape. Ecol Appl.

[5] Krupke CH, Hunt GJ, Eitzer BD, Andino G, Given K (2012). Multiple routes of pesticide exposure for honey bees living near agricultural fields. PLoS One.

[6] Mullins J, Emberlin J (1997). Sampling pollens. J Aerosol Sci.

[7] Keller A, Danner N, Grimmer G, Ankenbrand M, von der Ohe K, von der Ohe W (2015). Evaluating multiplexed next-generation sequencing as a method in palynology for mixed pollen samples. Plant Biol.

[8] Galimberti A, De Mattia F, Bruni I, Scaccabarozzi D, Sandionigi A, Barbuto M (2014). A DNA barcoding approach to characterize pollen collected by honeybees. PLoS One.

[9] Bruni I, Galimberti A, Caridi L, Scaccabarozzi D, De Mattia F, Casiraghi M (2015). A DNA barcoding approach to identify plant species in multiflower honey. Food Chem.

[10] Parducci L, Suyama Y, Lascoux M, Bennett KD (2005). Ancient DNA from pollen: a genetic record of population history in Scots pine. Mol Ecol.

[11] Bennett KD, Parducci L (2006). DNA from pollen: principles and potential. Holocene.

[12] Wilson EE, Sidhu CS, LeVan KE, Holway DA (2010). Pollen foraging behaviour of solitary Hawaiian bees revealed through molecular pollen analysis. Mol Ecol.

[13] Richardson RT, Lin C-H, Sponsler DB, Quijia JO, Goodell K, Johnson RM (2015). Application of ITS2 metabarcoding to determine the provenance of pollen collected by honey bees in an agroecosystem. Appl Plant Sci.

[14] Kraaijeveld K, de Weger LA, Ventayol García M, Buermans H, Frank J, Hiemstra PS (2015). Efficient and sensitive identification and quantification of airborne pollen using next-generation DNA sequencing. Mol Ecol Resour.

[15] Valentini A, Miquel C, Taberlet P (2010). DNA barcoding for honey biodiversity. Diversity.

[16] Kozich JJ, Westcott SL, Baxter NT, Highlander SK, Schloss PD (2013). Development of a dual-index sequencing strategy and curation pipeline for analyzing amplicon sequence data on the MiSeq Illumina sequencing platform. Appl Environ Microbiol.

[17] Chen S, Yao H, Han J, Liu C, Song J, Shi L (2010). Validation of the ITS2 region as a novel DNA barcode for identifying medicinal plant species. PLoS One.

[18] Gathmann A, Tscharntke T (2002). Foraging ranges of solitary bees. J Anim Ecol.

[19] Praz CJ, Müller A, Dorn S (2008). Host recognition in a pollen-specialist bee: evidence for a genetic basis. Apidologie.

[20] Edgar RC (2013). UPARSE: highly accurate OTU sequences from microbial amplicon reads. Nat Methods.

[21] Wang Q, Garrity GM, Tiedje JM, Cole JR (2007). Naive Bayesian classifier for rapid assignment of rRNA sequences into the new bacterial taxonomy. Appl Environ Microbiol.

[22] White TJ, Bruns T, Lee S, Taylor JW, Innis MA, Gelfand DH, Sninsky JJ, White TJ (1990). Amplification and direct sequencing of fungal ribosomal RNA genes for phylogenetics. PCR protocols: a guide to methods and applications.

[23] Fierer N, Hamady M, Lauber CL, Knight R (2008). The influence of sex, handedness, and washing on the diversity of hand surface bacteria. Proc Natl Acad Sci USA.

[24] Caporaso JG, Kuczynski J, Stombaugh J, Bittinger K, Bushman FD, Costello EK (2010). QIIME allows analysis of high-throughput community sequencing data. Nat Methods.

[25] Edgar RC (2010). Search and clustering orders of magnitude faster than BLAST. Bioinformatics.

[26] R Core Team (2014) R: A language and environment for statistical computing. Vienna, Austria. http://www.R-project.org/

[27] McMurdie PJ, Holmes S (2013). Phyloseq: an R package for reproducible interactive analysis and graphics of microbiome census data. PLoS One.

[28] Dixon P (2003). VEGAN, a package of R functions for community ecology. J Veg Sci.

[29] Keller A, Schleicher T, Schultz J, Müller T, Dandekar T, Wolf M (2009). 5.8S-28S rRNA interaction and HMM-based ITS2 annotation. Gene.

[30] Koetschan C, Förster F, Keller A, Schleicher T, Ruderisch B, Schwarz R (2010). The ITS2 Database III–sequences and structures for phylogeny. Nucleic Acids Res.

[31] Benson DA, Cavanaugh M, Clark K, Karsch-Mizrachi I, Lipman DJ, Ostell J (2013). GenBank. Nucleic Acids Res.

[32] Sayers EW, Barrett T, Benson DA, Bolton E, Bryant SH, Canese K (2011). Database resources of the national centre for biotechnology information. Nucleic Acids Res.

[33] Altschul SF, Gish W, Miller W, Myers EW, Lipman DJ (1990). Basic local alignment search tool. J Mol Biol.

[34] Soininen EM, Valentini A, Coissac E, Miquel C, Gielly L, Brochmann C (2009). Analysing diet of small herbivores: the efficiency of DNA barcoding coupled with high-throughput pyrosequencing for deciphering the composition of complex plant mixtures. Front Zool.

[35] Valentini A, Miquel C, Nawaz MA, Bellemain E, Coissac E, Pompanon F (2009). New perspectives in diet analysis based on DNA barcoding and parallel pyrosequencing: the trnL approach. Mol Ecol Resour.

[36] Gugerli F, Parducci L, Petit RJ (2004). Ancient plant DNA: review and prospects. New Phytol.

[37] Behling H, Pillar VD, Orlóci L, Bauermann SG (2004). Late Quaternary Araucaria forest, grassland (Campos), fire and climate dynamics, studied by high-resolution pollen, charcoal and multivariate analysis of the Cambará do Sul core in southern Brazil. Palaeogeogr Palaeoclimatol Palaeoecol.

[38] Davies AL, Tipping R (2004). Sensing small-scale human activity in the palaeoecological record: fine spatial resolution pollen analyses from Glen Affric, northern Scotland. Holocene.

[39] Woolfe M, Primrose S (2004). Food forensics: using DNA technology to combat misdescription and fraud. Trends Biotechnol.

