# Supplementary material for: Increased efficiency in identifying mixed pollen samples by meta-barcoding with a dual-indexing approach
Source: BMC Ecol. 2015 Jul 22;15:20. doi: 10.1186/s12898-015-0051-y (PMC4509727; doi:10.1186/s12898-015-0051-y)
Supplement: Additional file 1: — Plant species documented near solitary bee nest sites. [file 12898_2015_51_MOESM1_ESM.pdf]

Plant Species documented near solitary bee nest sites

No.	Species
1	*Abies spp.*
2	*Acer campestre*
3	*Acer spp.*
4	*Achillea millefolium*
5	*Achillea spp.*
6	*Acinos arvensis*
7	*Actaea spicata*
8	*Aegopodium podagraria*
9	*Aesculus spp.*
10	*Agrimonia eupatoria*
11	*Ajuga genevesis*
12	*Ajuga pyramidalis*
13	*Ajuga reptans*
14	*Allaria petiolata*
15	*Allium spp.*
16	*Allium ursinum*
17	*Alnus spp.*
18	*Anagallis arvensis*
19	*Anagallis foemina*
20	*Anemone ranunculoides*
21	*Anemone spp.*
22	*Anemone sylvestris*
23	*Anthemis tinctoria*
24	*Anthericum ramosum*
25	*Anthriscus sylvestris*
26	*Anthyllis vulneraria*
27	*Aquilegia vulgaris*
28	*Arctium lappa*
29	*Arctium tomentosum*
30	*Arnica spp.*
31	*Aster amellus*
32	*Aster linosyris*
33	*Ballota nigra*
34	*Barbarea vulgaris*
35	*Bellis perennis*
36	*Berberis vulgaris*
37	*Betula spp.*
38	*Brassica napus*
39	*Bryonia dioica*
40	*Bunias orientalis*
41	*Bupleurum falcatum*
42	*Calystegia sepium*
43	*Campanula glomerata*
44	*Campanula patula*

45 *Campanula persificolia*
46 *Campanula rapunculoides*
47 *Campanula rotundifolia*
48 *Campanula spp.*
49 *Campanula trachelium*
50 *Capsella bursa-pastoris*
51 *Cardamine pratensis*
52 *Carlina vulgaris*
53 *Caronum carvi*
54 *Carpinus spp.*
55 *Centaurea cyanus*
56 *Centaurea jacea*
57 *Centaurea montana*
58 *Centaurea scabiosa*
59 *Centaurea spp.*
60 *Centaurium erythraea*
61 *Cephalanthera rubra*
62 *Cephalanthera spp.*
63 *Cerastium arvense*
64 *Chelidonium majus*
65 *Cichorium intybus*
66 *Cirsium acaule*
67 *Cirsium arvense*
68 *Cirsium eriophorum*
69 *Cirsium spp.*
70 *Cirsium vulgare*
71 *Clematis vitalba*
72 *Clinopodium vulgare*
73 *Colchicum autumnale*
74 *Consolida regalis*
75 *Convallaria majalis*
76 *Convolvulus arvensis*
77 *Cornus mas*
78 *Cornus sanguinea*
79 *Coronilla spp.*
80 *Corydalis cava*
81 *Corylus avellana*
82 *Crataegus leavigata*
83 *Crataegus monogyna*
84 *Crataegus spp.*
85 *Crepis biennis*
86 *Crepis spp.*
87 *Delphinium spp.*
88 *Dianthus carthusianorum*
89 *Digitalis grandiflora*
90 *Dipsacus fullonum*
91 *Echinops sphaerocephalus*

92 *Echium vulgare*
93 *Epilobium angustifolium*
94 *Epilobium hirsutum*
95 *Erigeron annuus*
96 *Erodium cicutarium*
97 *Erophila verna*
98 *Eryngium spp.*
99 *Euonymus europaeus*
100 *Euphorbia cyparissias*
101 *Euphorbia falcata*
102 *Euphorbia helioscopia*
103 *Euphorbia spp.*
104 *Fagus spp.*
105 *Ficaria verna (Ranunculus ficaria)*
106 *Filago spp.*
107 *Filipendula ulmaria*
108 *Forsythia vahl*
109 *Fragaria vesca*
110 *Frangula alnus*
111 *Fraxinus excelsior*
112 *Fumaria officinale*
113 *Gagea lutea*
114 *Galeopsis angustifolium*
115 *Galeopsis spp.*
116 *Galium aparine*
117 *Galium odoratum*
118 *Galium verum*
119 *Gallium mollugo*
120 *Genista tinctoria*
121 *Gentiana ciliata*
122 *Geranium pratense*
123 *Geranium pyrenaicum*
124 *Geranium robertanium*
125 *Geranium sanguineum*
126 *Geranium spp.*
127 *Geum urbanum*
128 *Glechoma hederacea*
129 *Helianthemum nummularium*
130 *Helianthemum spp.*
131 *Helianthenum apeninum*
132 *Helianthus annuus*
133 *Hippocrepis comosa*
134 *Hiracium murorum*
135 *Hiracium spp.*
136 *Hypericum spp.*
137 *Ilex aquifolium*
138 *Impatiens parviflora*

139 *Inula salicina*
140 *Iris spp.*
141 *Isatis tinctoria*
142 *Juglans regia*
143 *Knautia arvensis*
144 *Laburnum anagyroides*
145 *Lactuca serriola*
146 *Lamium album*
147 *Lamium amplexicaule*
148 *Lamium galeobdolon*
149 *Lamium maculatum*
150 *Lamium purpureum*
151 *Lamium spp.*
152 *Lapsana communis*
153 *Larix spp.*
154 *Lathyrus latifolius*
155 *Lathyrus pratensis*
156 *Lathyrus spp.*
157 *Lathyrus sylvestris*
158 *Lathyrus tuberosus*
159 *Lathyrus vernus*
160 *Leucanthemum vulgare*
161 *Ligustrum spp.*
162 *Linaria vulgaris*
163 *Linda spp.*
164 *Linum spp.*
165 *Lonicera periclymen*
166 *Lonicera tatarica*
167 *Lonicera xylosteum*
168 *Lotus corniculatus*
169 *Lunaria rediviva*
170 *Lupinus spp.*
171 *Lythrum salicaria*
172 *Malus spp.*
173 *Malva spp.*
174 *Matricaria chamomilla*
175 *Mediago lupulina*
176 *Medicago sativa*
177 *Medicago spp.*
178 *Melampyrum arvense*
179 *Melilotus albus*
180 *Melilotus officinale*
181 *Mespilus germanica*
182 *Muscari neglectum*
183 *Mycelis muralis*
184 *Myosotis arvensis*
185 *Myosotis spp.*

186 *Oenothera biennis*
187 *Onobrychis viciifolia*
188 *Ononis repens*
189 *Ononis spinosa*
190 *Ophrys apifera*
191 *Orchis militaris*
192 *Orchis purpurea*
193 *Origanum vulgare*
194 *Papaver roheas*
195 *Pastinaca sativa*
196 *Phacelia spp.*
197 *Picea spp.*
198 *Picris hieracioides*
199 *Pinus spp.*
200 *Plantago lanceolata*
201 *Plantago major*
202 *Planthera bifolia*
203 *Platanthera chlorantha*
204 *Polygala amara*
205 *Potentilla reptans*
206 *Primula spp.*
207 *Prunella grandiflora*
208 *Prunella vulgaris*
209 *Prunus avium*
210 *Prunus mahaleb*
211 *Prunus padus*
212 *Prunus spinosa*
213 *Prunus spp.*
214 *Pulsatilla vulgaris*
215 *Pyrus spp.*
216 *Quercus spp.*
217 *Rannuculus spp.*
218 *Rhinanthus alectorolophus*
219 *Rhinanthus spp.*
220 *Robinia pseudoacacia*
221 *Rosa spp.*
222 *Rubus spp.*
223 *Salix spp.*
224 *Salvia pratense*
225 *Salvia verticillata*
226 *Sambucus spp.*
227 *Saponaria spp.*
228 *Saxifraga granulata*
229 *Scilla bifolia*
230 *Securigera varia*
231 *Sedum acre*
232 *Sedum rupestre*

233 *Sedum spp.*
234 *Sedum spurium*
235 *Senecio jacobea*
236 *Senecio ovatus*
237 *Senecio spp.*
238 *Senecio vulgaris*
239 *Silene dioica*
240 *Silene flos-cuculi*
241 *Silene latifolia*
242 *Silene nutans*
243 *Silene spp.*
244 *Silene viscaria*
245 *Silene vulgaris*
246 *Sinapis arvensis*
247 *Solanum nigrum*
248 *Solidago virgaurea*
249 *Sonchus asper*
250 *Sonchus spp.*
251 *Sorbus aucuparia*
252 *Sorbus torminales*
253 *Stachys officinalis (Betonica officinalis)*
254 *Stachys palustris*
255 *Stachys recta*
256 *Stachys spp.*
257 *Stellaria holostea*
258 *Stellaria media*
259 *Stellaria spp.*
260 *Symphytum officinale*
261 *Syringa vulgaris*
262 *Tanacetum corymbosum*
263 *Tanacetum parthenium*
264 *Tanacetum vulgare*
265 *Taraxacum officinale*
266 *Taraxacum spp.*
267 *Tetragonolobus maritimus*
268 *Teucrium botrys*
269 *Teucrium chamaedrys*
270 *Thlaspi perfoliatum*
271 *Thymus pulegoides*
272 *Tilia spp.*
273 *Tragopogon pratense*
274 *Trifolium spp.*
275 *Tripleurospermum maritimum; syn. perforatum*
276 *Trollius spp.*
277 *Tulipa spp.*
278 *Tussilago farfara*
279 *Valeriana officinalis*

280 *Verbascum lynchnitis*
281 *Veronica chamydris*
282 *Viburnum lantana*
283 *Viburnum opulus*
284 *Vicia cracca*
285 *Vicia sepium*
286 *Vinca min*
287 *Vincetoxicum hirundinaria*
288 *Viola arvensis*
289 *Viola reichenbachiana*
290 *Viola spp.*
291 *Viola tricolor*
292 *Zea mays*
