# Supplementary material for: Increased efficiency in identifying mixed pollen samples by meta-barcoding with a dual-indexing approach
Source: BMC Ecol. 2015 Jul 22;15:20. doi: 10.1186/s12898-015-0051-y (PMC4509727; doi:10.1186/s12898-015-0051-y)
Supplement: Additional file 2: — Table S1. Comparison of the number of genera per order for all orders. [file 12898_2015_51_MOESM2_ESM.pdf]

Table S1: Comparison of the number of genera per order for all orders.

Order	TaxID	Genera old	Genera new
Acorales	91812	1	1
Acrosiphoniales	66259	0	3
Alismatales	16360	24	69
Andreaeales	13794	0	1
Anthocerotales	13810	0	1
Apiales	4036	319	406
Aquifoliales	91883	3	4
Araucariales	1446378	10	22
Arecales	40551	44	70
Asparagales	73496	628	837
Asterales	4209	887	1211
Austrobaileyales	82956	3	3
Bartramiales	1034061	0	8
Boraginales	1538097	69	107
Brassicales	3699	148	360
Bruniales	703243	1	12
Bryales	3226	14	17
Bryopsidales	33104	2	10
Bryoxiphiales	404270	0	1
Buxales	280577	3	6
Buxbaumiales	404267	0	1
Canellales	71187	13	13
Caryophyllales	3524	216	422
Celastrales	233875	47	78
Ceratophyllales	91811	1	1
Chaetophorales	31299	1	10
Charales	204509	1	2
Chlamydomonadales	3042	23	34
Chloranthales	261008	2	3
Chlorellales	35460	11	28
Chlorocystidales	578868	1	1
Chlorodendrales	35426	1	2
Chlorosarcinales	138177	0	1
Cladophorales	3183	1	18
Commelinales	4739	0	1
Cornales	41934	6	14
Crossosomatales	232392	4	5
Cucurbitales	71239	68	85
Cupressales	1446379	26	31
Cyatheales	693763	0	4
Cycadales	3297	10	10

Table S1: Comparison of the number of genera per order for all orders.

Order	TaxID	Genera old	Genera new
Dasycladales	3134	0	1
Dendrocerotales	400689	0	4
Desmidiales	131210	2	8
Dicranales	3219	11	35
Dilleniales	403665	0	2
Dioscoreales	40548	4	12
Dipsacales	4199	26	41
Dolichomastigales	1525213	1	2
Ephedrales	3385	0	1
Equisetales	3255	0	1
Ericales	41945	173	285
Fabales	72025	413	524
Fagales	3502	37	38
Fossombroniales	186784	3	1
Funariales	3215	1	7
Garryales	91889	2	3
Gentianales	4055	401	624
Geraniales	41943	3	15
Gigaspermales	1031676	0	3
Ginkgoales	3308	0	1
Gnetales	3378	1	1
Grimmiales	64936	5	8
Gunnerales	232382	1	1
Hedwigiales	114664	0	2
Hookeriales	65545	13	38
Hypnales	13798	198	261
Hypnodendrales	480566	0	3
Ignatiales	231076	0	1
Isoetales	13836	1	1
Jungermanniales	3199	26	69
Klebsormidiales	3172	4	5
Lamiales	4143	457	702
Laurales	3432	47	65
Liliales	4667	18	43
Lycopodiales	3249	0	4
Magnoliales	3400	4	14
Malpighiales	3646	161	257
Malvales	41938	145	180
Mamiellales	13792	3	5
Marchantiales	28908	2	8
Metzgeriales	34158	3	3

Table S1: Comparison of the number of genera per order for all orders.

Order	TaxID	Genera old	Genera new
Microthamniales	42111	4	6
Monomastigales	1525214	1	1
Myrtales	41944	152	252
Notothyladales	400691	0	2
Nymphaeales	261007	3	8
Oedogoniales	35490	3	3
Orthotrichales	64937	0	4
Oxalidales	71243	6	18
Pallaviciniales	402723	5	6
Pandanales	40550	1	9
Pedinomonadales	35423	0	1
Pelliales	400718	1	1
Pinales	1446380	10	11
Piperales	16736	7	12
Poales	38820	366	570
Polypodiales	3268	4	10
Polytrichales	3210	9	10
Porellales	186798	45	68
Pottiales	38585	24	46
Prasinococcales	485343	0	1
Prasiolales	135250	0	1
Proteales	232378	70	70
Psilotales	3237	1	1
Ptilidiales	984499	1	1
Ptychomniales	404314	1	4
Pyramimonadales	38834	0	2
Ranunculales	41768	78	137
Rhizogoniales	114662	1	2
Rosales	3744	110	219
Salviniales	74353	0	3
Santalales	41947	13	62
Sapindales	41937	171	240
Saxifragales	41946	71	105
Schizaeales	693762	0	1
Scouleriales	404269	0	2
Selaginellales	3244	1	1
Solanales	4069	67	84
Sphaerocarpales	37407	0	2
Sphaeropleales	35491	18	43
Sphagnales	13802	0	2
Splachnales	64938	0	4

Table S1: Comparison of the number of genera per order for all orders.

Order	TaxID	Genera old	Genera new
Takakiales	70832	0	1
Tetraphidales	37417	1	1
Tetrasporales	31305	2	6
Timmiales	114659	0	1
Trentepohliales	35443	0	2
Trochodendrales	400839	1	2
Ulotrichales	31306	0	11
Ulvales	3113	8	14
Vitales	403667	1	7
Welwitschiales	3374	0	1
Zingiberales	4618	78	87
Zygnematales	3176	0	2
Zygophyllales	403666	8	11
Coleochaetales	204510	1	0
