# Supplementary material for: Increased efficiency in identifying mixed pollen samples by meta-barcoding with a dual-indexing approach
Source: BMC Ecol. 2015 Jul 22;15:20. doi: 10.1186/s12898-015-0051-y (PMC4509727; doi:10.1186/s12898-015-0051-y)
Supplement: Additional file 3: — Table S2. Comparison of the number of sequences per group for selected taxonomic groups. [file 12898_2015_51_MOESM3_ESM.pdf]

Table S2: Comparison of the number of sequences per group for selected taxonomic groups.

Group	old	new
Vitaceae	1	62
Heracleum	80	414
Carduus	10	19
Phacelia	34	176
Convolvulus	161	230
Helianthus	72	80
